# Upgrading the Transdermal Biomedical Capabilities of Thyme Essential Oil Nanoemulsions Using Amphiphilic Oligochitosan Vehicles

**DOI:** 10.3390/pharmaceutics14071350

**Published:** 2022-06-25

**Authors:** Ali M. Nasr, Yasmin I. Mortagi, Nashwa H. Abd Elwahab, Mohammad Y. Alfaifi, Ali A. Shati, Serag Eldin I. Elbehairi, Reda F. M. Elshaarawy, Islam Kamal

**Affiliations:** 1 Department of Pharmaceutics, Faculty of Pharmacy, Port Said University, Port Said 42526, Egypt; a.nasr@pharm.psu.edu.eg (A.M.N.); islamkamal@pharm.psu.edu.eg (I.K.); 2 Department of Pharmaceutics, Faculty of Pharmacy, Sinai University, Alarish 45511, Egypt; yasmin.mohamed@su.edu.eg; 3 Department of Pharmaceutics and Industrial Pharmacy, Faculty of Pharmacy, Sinai University-Kantara Branch, Ismailia 41636, Egypt; nashwa.abdelwahab@su.edu.eg; 4 Biology Department, Faculty of Science, King Khalid University, Abha 9004, Saudi Arabia; alfaifi@kku.edu.sa (M.Y.A.); aaalshati@kku.edu.sa (A.A.S.); serag@kku.edu.sa (S.E.I.E.); 5 Cell Culture Lab, Egyptian Organization for Biological Products and Vaccines (VACSERA Holding Company), 51 Wezaret El-Zeraa St., Agouza, Giza 12654, Egypt; 6 Department of Chemistry, Faculty of Science, Suez University, Suez 43533, Egypt; 7 Institut für Anorganische Chemie und Strukturchemie, Heinrich-Heine Universität Düsseldorf, 40225 Düsseldorf, Germany

**Keywords:** thyme oil multilayer nanoemulsions, release kinetics, transdermal delivery, anti-inflammatory, anti-melanoma

## Abstract

(1) Background: *Thymus vulgaris* L. (thyme) essential oil (TEO) has gained much attention because of its long history of medicinal usage. However, the lack of precise chemical profiling of the TEO and methods to optimize the bioactivity and delivery of its constituents has hampered its research on quality control and biological function; (2) Methods: The current study aimed to analyze the TEO’s chemical composition using the GC-MS method and identify its key components. Another objective of this work is to study the impact of the protective layer of amphiphilic oligochitosan (AOC) on the physicochemical stability and transdermal potentials of TEO multilayer nanoemulsions formulated by the incorporation of TEO, Tween80, lecithin (Lec), and AOC; (3) Results: The AOC protective layer significantly improved the stability of TEO-based NEs as revealed by the constancy of their physicochemical properties (particle size and zeta potential) during storage for a week. Excessive fine-tuning of thyme extract NEs and the AOC protective layer’s persistent positive charge have been contributed to the thyme extract’s improved anti-inflammatory, transdermal, and anti-melanoma potentials; (4) Conclusions: the AOC-coated NEs could offer novel multifunctional nanoplatforms for effective transdermal delivery of lipophilic bioactive materials.

## 1. Introduction

In recent years, the use of nanoemulsion-based systems to deliver lipophilic bioactive ingredients such as vaccines, proteins, vitamins, essential oils [1], preservatives, antimicrobial [2], anticancer [3], antiviral, anti-inflammatory, ophthalmic [4], and Alzheimer’s drugs [5] has attracted great interest. Embedding bioactive ingredients into the nanoemulsions (NEs) enhances their physicochemical stability, imparts functional potentials, and increases bioavailability as well as transdermal permeability, all of which affect the nanoemulsion’s end-use properties in medicinal and cosmetic applications [6]. Although NEs are stabilized using surfactants, nevertheless, the stability of these systems may be decreased with time, resulting in their sedimentation, creaming, coagulation, and flocculation, which consequently limit their functional potentials [6]. 

Modifying the surface properties of emulsions by coating them with an oppositely charged biopolymer (proteins, lipids, or polysaccharides) offers a promising strategy for improving the stability and applicability of emulsions. When using this approach, double-layered emulsions have been formed in which the ionic emulsifier is initially used to create a “primary” emulsion core, and then an oppositely charged biopolymer is adsorbed onto the droplet surfaces of the “primary” emulsion to create the secondary emulsifying layer [7]. Emulsion droplets could be prevented from aggregating by generating mutual electrostatic and steric repulsive interactions between emulsion droplets coated with charged biopolymer [8]. The amazing characteristics and potentials of chitosan (CS) [9,10] as well as its capability to form extremely stable CS-coated secondary emulsion systems via electrostatic interactions with anionic surfactants or biomacromolecules (sodium dodecyl sulfate (SDS), sodium stearoyl, lecithin, ε-polylysine (EPL), poly(lactic-co-glycolic) acid (PLGA), and others) [11,12,13], make it a very attractive platform to formulate stable nanoemulsion delivery systems (NEDSs). However, chitosan’s limited aqueous solubility has limited its widespread use in the formulation of NEs. Nevertheless, the ease of the surface functionalization of chitosan could prompt its aqueous solubility and open up new avenues for enhancing the biological effects of NEs. To name a few, carboxymethyl chitosan plays an important synergistic role in the formulation of clove essential oil (CEO) nanoemulsion by enhancing its physical stability and promoting its antimicrobial potential for prolonged meat preservation applications [14]. Another study revealed that using amphiphilic chitosan for nanoencapsulation lemongrass essential oil (LEO) not only enhances the physical stability of the obtained LEO-based NEs but also increases their antimicrobial activity [15].

Among essential oils (EOs), thyme essential oil (TEO) extracted from *Thymus vulgaris* L. (thyme) has attracted the attention of many researchers due to its long history of pharmacological uses as well as its organoleptic properties. Where it has a long history of traditional use in the Mediterranean region for treating respiratory tract infections, whooping and spasmodic coughs, sore throats, colds, tonsillitis, dyspepsia, colic, and intestinal infections and infestations [16]. Recently, TEO was found to have high antidiabetic activity through inhibition of α-glucosidase enzyme and suitable anticancer activity against MCF-7 cells through DNA fragmentation and cell apoptosis [17]. These beneficial effects are mostly due to the presence of numerous identified chemotypes bioactive ingredients. The dominant chemotypes are thymol, linalool, carvacrol, geraniol, sabinene hydrate, α-terpineol, 1,8-cineole, and *p*-cymene [18]. Recent studies demonstrated the effective roles of chitosan/TEO nanoemulsions in antimicrobial packaging and increasing the shelf-life of the refrigerated pork [19], antimicrobial delivery systems in foods [20], preserving fruit quality during ripening [21], controlling anthracnose in mango fruits and prolonging their shelf-life [22], and inhibiting microbial growth from improving the shelf-life of Karish cheese [23]. So far, no report has been published on the formulation and use of CS-based nanoemulsion loaded with TEO, targeting improving the anticancer, anti-inflammatory, and transdermal characteristics of thyme essential oil. 

These outstanding facts, coupled with our continuous interest in developing novel, safe pharmacological systems [24,25,26], motivated us to evaluate the ability of new amphiphilic oligochitosan (AOC) to stabilize VOO nanoemulsion as well as to improve the bioavailability, antioxidant, and anticancer properties of the olive extract. As far as we know, the nanoemulsions targeted in this study will be the first multilayer nanoemulsion systems comprising thyme oil and Tween80-lecithin as a primary layer and AOC as a secondary protective layer.

## 2. Materials and Methods

The electronic supplementary information (**ESI†**) provided details for the specifications of chemicals used in this study as well as their suppliers. In addition, the analytical techniques used to comprehensively characterize the prepared materials are described in the **ESI†**. Moreover, the glycidylammonium chloride salt, oligochitosan chitosan (OC), and amphiphilic oligochitosan (AOC) were obtained from crab wastes as described in our previous work [24,25].

### 2.1. TEO Extraction

#### 2.1.1. Plant Sampling

The fresh aerial parts of *Thymus vulgaris* were purchased from the Center of Medicinal and Aromatic Plants Research, Faculty of Pharmacy; Cairo University, Egypt. The plant samples were immediately frozen after purchase until they were used. 

#### 2.1.2. Essential Oil Extraction

TEO was extracted by hydrodistillation for 3 h using a Clevenger-type apparatus according to the protocol adapted by the Egyptian Pharmacopoeia [27]. The upper oil layer was isolated, dried over sodium sulfate anhydrous, and then was reserved in sealed air-tight glass vials in a refrigerator at 4 °C until used. 

#### 2.1.3. GC-MS Analysis

The chemical composition of the extracted TEO was investigated by the gas chromatography-mass spectrometry (GC-MS) technique using a TRACE GC Ultra Gas Chromatograph (THERMO Scientific Corp., USA) and a thermo MS detector (ISQ Single Quadrupole MS). A capillary column TR-5 MS (30 m × 0.32 mm ID, 0.25 μm film thickness) was used to fractionate the oil samples. Operating conditions were as follows: Split ratio, 1:10; He gas flow 1.0 mL/min; Injection volume 1.0 μL; Column temperature maintained at 40 °C for 1 min, then raised at 4 °C/min to 180 °C and held for 1 min, then raised at 4 °C/min to 240 °C at 1 °C/min; Injector, transfer line, and detector temperatures were 210 °C, 270 °C, and 210 °C, respectively. The isolated fractions were diluted with hexane (1:10 hexane, *v*/*v*), and then 1 μL of the mixtures were injected, and mass spectra were obtained by electron ionization (EI) at 70 eV using a spectral range of *m/z* 40−450. To identify the chemical ingredients of the TEO, the chromatogram peaks were first de-convoluted with AMDIS software (www.amdis.net, accessed on 16 October 2021) and identified by matching the mass spectral patterns and retention indices of these ingredients to those of standard compounds or by comparing their mass spectra to those in the Wiley spectral library collection and NSIT library database [28]. In addition, the Kováts indices were calculated and compared to published retention indices. Compounds were quantified based on their area in the chromatogram.

#### 2.1.4. Preparation of NEs

The previously reported protocol [29] was used to prepare the desired NEs. Initially, stock solutions of OC/AOC (2%, *w*/*v*) and lecithin (10%, *w*/*v*) were prepared by dissolving OC/AOC and lecithin in 1% (*v*/*v*) acetic acid and DIW, respectively. The target NEs were then prepared in two successive steps. 

In the first step, the low-energy emulsification process was applied to prepare the primary NEs by the addition of an oily component (TEO-Tween80) to the aqueous component (lecithin (Lec) solution) under vigorous stirring. The emulsification process was set up to achieve a final composition of primary nanoemulsions of 1% TEO, 9% Tween80, and 0.5% Lec. In the initial phase, an oil phase was prepared by mixing TEO extract with Tween80 under stirring (500 rpm) for 10 min. The combined oil phase was dropwise added within 10 min into an aqueous phase containing lecithin under vigorous stirring. The obtained primary nanoemulsions were diluted with DIW (1:1) to obtain bare NEs (**NE1**) with a final composition of TEO, Tween80, and Lec of 1%, 9%, and 0.5%, respectively.

In the second step, the secondary NEs were prepared by coating the primary bare NEs with OC or AOC through the electrostatic deposition of positively charged biopolymers onto the surface of negatively charged lecithin molecules. To determine the optimum dose of CS to be deposited, solutions of serial concentrations of OC (0.0, 0.03, 0.06, 0.125, 0.25 and 0.5% wt%) were prepared in 0.1 M acetic acid/DIW. Thereafter, 7 mL of each OC solution was rapidly added to 7 mL of 7 mL primary NEs under vigorous stirring to obtain secondary nanoemulsions with final weight ratios of 16:1, 8:1, 4:1, 2:1, and 1:1 of lecithin to OC (**OC-NE1** to **OC-NE5**, respectively) (see Table S1, **ESI†**). To ensure homogeneous CS deposition, the obtained mixture was homogenized by a high-speed homogenizer (HSH, HG-15-A, DAIHAN Co., Seoul, Korea) at 5000 rpm for 10 min, followed by sonication using a probe-type ultrasonicator (US, Sonic & Materials, USA) for 5 min in an ice-water bath. The obtained OC-Lec-NEs were kept at 4 °C until use. On the other hand, to investigate the impact of the AOC quaternization degree (QD) on the stability of its respective secondary NEs, the primary NE was diluted with equi-volume solutions of the as-prepared AOC with different QD%, using the aforesaid protocol to obtain the secondary AOC-Lec-NEs (AOC-**NE1** to AOC-**NE5**). 

### 2.2. Encapsulation Efficiency (EE) and Oil Loading (OL)

The percentage of TEO entrapped into the nanodroplets (encapsulation efficiency (EE)) and the concentration of the oil loaded per unit weight of nanoemulsion (encapsulation efficiency (OL)) were quantified spectrophotometrically at 274 nm, using a previously reported protocol [30]. In brief, an aliquot of 500 μL of nanoemulsion was solubilized in 5 mL of a mixed-solvent system (acetonitrile-water, 0.95:1 *v*/*v*), and the obtained mixture was kept at room temperature for 48 h to ensure the release of entrapped oil into the solution. After centrifugation at 10,000 rpm for 5 min, the liberated oil content was estimated in the supernatant spectrophotometrically at 274 nm. All experiments were carried out in triplicates, and the results were presented as mean ± standard deviation (SD). The EE and OL of TEO were calculated using the following equations (Equations (1) and (2)) [30]:

(1) $$\mathrm{EE}\%=\frac{\mathrm{Amount}\;\mathrm{of}\;\mathrm{TEO}\;\mathrm{entrapped}}{\mathrm{Initial}\;\mathrm{amount}\;\mathrm{of}\;\mathrm{TEO}}\times 100$$

(2) $$\mathrm{OL}\%=\frac{\mathrm{Amount}\;\mathrm{of}\;\mathrm{TEO}\;\mathrm{entrapped}}{\mathrm{Initial}\;\mathrm{amount}\;\mathrm{of}\;\mathrm{NE}}\times 100$$

### 2.3. Ex Vivo Skin Permeability Study

The ability of nanoemulsions with the highest in vitro release profiles to cross the skin barrier was studied using rat skin (RS) as a natural membrane. The protocol used to perform ex vivo skin permeation studies was approved by the National Hepatology & Tropical Medicine Research Institute’s Ethics Committee (NHTMRI), Egypt. 

#### 2.3.1. Permeability Experiment 

White albino rats (3–4 months old, 250–350 g weight) were obtained from the animal house of the National Research Center (NRC), Giza, Egypt. After sacrificing rats, the abdominal skin of a fresh rat was excised, and the skin hair was discarded using an electric clipper. The skin was then cleaned from the subcutaneous tissues surgically. The skin was submerged in a normal saline solution (0.9% NaCl), with the stratum corneum (SC) facing up for one hour, and then washed with fresh saline solution to remove the extraneous debris and leachable enzymes. Finally, the skin was stored in a freezer until use. The Franz diffusion cell (FDC) effective diffusional area of 2.44 cm^2^ was used to carry out the ex vivo skin permeability studies. The excised rat skin was placed between the donor and receiver compartments of the FDC, with its SC facing the donor chamber. The receiving compartment was filled with PBS (pH 7.4) and kept under continuous stirring (500 rpm) at 37 ± 0.5 °C. One milliliter of each nanoemulsion sample was placed in the donor chamber, which was then covered with Parafilm. Samples were withdrawn from the receptor fluid in aliquots of 0.5 mL at regular intervals (1, 2, 4, 8, 12, 16, 24, and 48 h), filtered through a 0.45 mm membrane filter, and analyzed spectrophotometrically at λ_max_ 274 nm for TEO content in skin leachables, to evaluate the permeation kinetics, and replenished by equivalent volumes of fresh PBS. The cumulative quantity of TEO permeated through the skin (Q_n_, mg/cm^2^) was quantified using the following equation (Equation (3)) [31]:

(3) $$Q_{n}=\frac{C_{t}V_{r}+\sum_{i\;=0}^{t-1}C_{i}V_{s}\;}{A}$$

where C_t_ and C_i_ are the TEO concentrations in the receptor compartment at sampling times t and ith (t − 1), respectively, V_r_ and vs. are the volumes of the receptor compartment solution (12 mL) and withdrawn sample (0.5 mL), respectively, and A is the effective diffusional area. 

#### 2.3.2. Permeability Data Analysis 

The cumulative amount of TEO TEO (Q_n_, mg/cm^2^) infiltrated through the albino rat skin was graphed versus the permeation time (t, h) for the TEO nanoemulsion. The permeability parameters, including steady-state transdermal flux (*J*_ss_, mg cm^–2^ h^–1^), permeability coefficient (*K*_p_, cm h^–1^), and enhancement ratio (*E*_r_), were calculated using the following equations (Equations (4)–(6)) [32]:

(4) $$J_{\mathrm{ss}}=\frac{\mathrm{Slope}\;\mathrm{of}\;\mathrm{the}\;\mathrm{linear}\;\mathrm{part}\;\mathrm{of}\;\mathrm{the}\;\mathrm{graph}\;}{\mathrm{Diffusion}\;\mathrm{cell}\;\mathrm{area}}$$

(5) $$K_{p}=\frac{J_{\mathrm{ss}}\;}{\mathrm{Initial}\;\mathrm{concentration}\;\mathrm{of}\;\mathrm{NE}}$$

(6) $$E_{r}=\frac{J_{\mathrm{ss}}\;\mathrm{for}\;\mathrm{NE}\;}{J_{\mathrm{ss}}\;\mathrm{for}\;\mathrm{control}\;\left(\mathrm{TEO}\right)}$$

### 2.4. In Vitro TEO Release Kinetics 

The in vitro release style of TEO from the new nanoemulsions (NE1 and AOC-NE3) was investigated in phosphate buffer saline (PBS) (pH ~7.4) following the previously reported protocol [33], with a slight modification. Briefly, an aliquot of 2 mL of nanoemulsion was diluted with 20 mL of PBS into a glass vial. The content was then stirred (200 rpm) at room temperature for 4 h. At definite time intervals, while stirring, a 2 mL aliquot of each sample was withdrawn and subjected to centrifugation (10,000 rpm), and the sample was replenished with an equal volume of fresh PBS. Thereafter, 1 mL of supernatant was submitted for TEO spectrophotometric analysis at 274 nm. The TEO release was calculated by the following equation (Equation (7)):

(7) $$\mathrm{Release}\left(\%\right)=\overset{t}{\underset{t=0}{\sum }}\frac{M_{t}}{M_{0}}\times 100$$

where M_t_ is the released amount of TEO at each sampling time, and M_0_ is the initial VOO amount encapsulated in the nanoemulsion.

### 2.5. Validation of UV-Vis Spectrophotometric Method for TEO Estimation 

Initially, scans were performed in the range of 200–400 nm using TEO stock solution in MeCN (1 mg/mL) to determine the wavelength that permits the maximum absorption of TEO in UV. Following that, ICH criteria [34] were used to carry out the technique validation and confirm its accuracy. The validity indices, including linearity, accuracy, quantification limit (QL), and detection limit (DL) were calculated from the slope (S) and standard deviation (SD) of the calibration curve of UV-absorbance against initial TEO concentrations in nanoemulsion at 274 nm using the following equations (Equations (8) and (9)) [34]:

(8) $$\mathrm{QL}=\frac{10\;\mathrm{SD}\;}{S}$$

(9) $$\mathrm{DL}=\frac{3.3\;\mathrm{SD}\;}{S}$$

### 2.6. In Vitro Anti-Inflammatory Activity 

The inhibitory effect of the most cytotoxic nanoemulsions on the level of the inflammatory mediator (nitric oxide, NO) produced by the murine macrophage-like RAW 264.7 cells was used to evaluate the anti-inflammatory activity of these NEs. To perform this test, we used a protocol that had been somewhat tweaked from a previous study [35]. In brief, DMEM media supplemented with penicillin G (100 U/mL), streptomycin (100 mg/mL), and 10% FCS was used to grow RAW 264.7 cells at 37 °C in a humidified atmosphere containing 5% CO_2_. They were then diluted in fresh medium after harvesting them with trypsin-EDTA. After that, 105 cells were sown into each well of a 96-well plate. After one day, the medium was subrogated with solutions of the tested samples at various concentrations blended with 1 μg/mL of LPS (Sigma-Aldrich, Schnelldorff, Germany) to stimulate RAW cells, and they were incubated for 48 h. The NO productivity was evaluated by measuring the cumulative nitrite concentration in the culture supernatant using a Griess colorimetric method at 540 nm. 

On the other hand, the toxicity of tested NEs toward RAW 264.7 cells was assessed using the MTT assay. Briefly, 20 μL of MTT solution in PBS (3 mg/mL) was added to the 48 h-incubated wells and then incubated for an additional 4 h. After incubation, the medium was replaced with DMSO to solubilize the formed formazan crystals. Thereafter, the microplate reader was used to measure the absorbance at 540 nm. Diclofenac was employed as a positive control.

### 2.7. Cytotoxicity Study 

#### 2.7.1. Cell Culture Protocol

The most serious type of skin cancer cell lines (melanoma, A375) and normal human skin fibroblast (HSF) cells were obtained from the American Type Culture Collection through Nawah-Scientific research center, Egypt. These cells were cultivated in the Dulbecco’s Modified Eagle’s Medium (DMEM) supplemented with 10% Fetal Bovine Serum (FBS), 10 μg/mL of insulin, and 1% penicillin G (100 U/mL)—streptomycin (100 mg/mL) at 37 °C in a humidified environment with 5% CO_2_. The culture medium was then carefully removed, and the cells were harvested as well as rinsed with Trypsin (0.25% *w*/*v*)—EDTA (0.53 mM) solution and re-suspended in a fresh growth medium. Finally, the cell cultures were incubated for 24 h at 37 °C. 

#### 2.7.2. MTT Assay

The ability of new bioactive materials to eliminate viable cells responsible for the reduction in MTT dye into purple formazan products is strongly correlated with their cytotoxicity. Therefore, the MTT assay has used trophotometric measurements to monitor the in vitro cytotoxicity of new nanoemulsions. In brief, 200 microliters of each cell culture were seeded into a 96-well plate at a cell density of 10^5^ cells/well, and the plate was incubated at 37 °C overnight. On the next day, the cells were treated with 200 μL of each nanoemulsion in serial concentrations and incubated for 48 h. The MTT solution (20 μL) was then added, followed by re-incubation for a further 4 h. After incubation, each well was discharged from the treating medium, and the formed formazan crystals were dissolved in 150 μL of DMSO. The plate was gently shaken in a gyratory shaker to promote the dissolution of formazan crystals before measuring absorbance spectrophotometrically at 540 nm. 

### 2.8. Statistical Analysis

All experiments were performed in triplicate, and the results were expressed as a mean ± standard deviation. The OriginPro 9.1 software was used to graph the obtained results. Mathematical treatment and statistical comparisons were performed using a one-way analysis of variance (ANOVA) test) (SPSS software v. 22, Chicago, IL, USA), followed by Tukey’s multiple comparison post-hoc test for pairwise comparisons. Differences with values of *p* < 0.05 were assigned as statistically significant.

## 3. Results and Discussion

### 3.1. Chemical Composition of TEO

The TEO was extracted from *Thymus vulgaris* L. using the traditional hydrodistillation (HD) technique. GC-MS chromatogram (Figure S1, **ESI†**) and Table 1 list the identified chemical ingredients in the TEO extracted from the thyme aerial parts by HD. The 49 compounds listed in Table 1 formulate 99.12% of the total GC chromatogram peak areas. The main components in the TEO are the oxygenated monoterpenes (Thymol, Carvacrol), sesquiterpene (Germacrene D), and oxygenated sesquiterpenes (α-Acorenol, D-Germacren-4-ol). Noteworthy, more than 75% of the ingredients of TEO are oxygenated compounds.

### 3.2. Synthesis Protocol

**Scheme 1:**
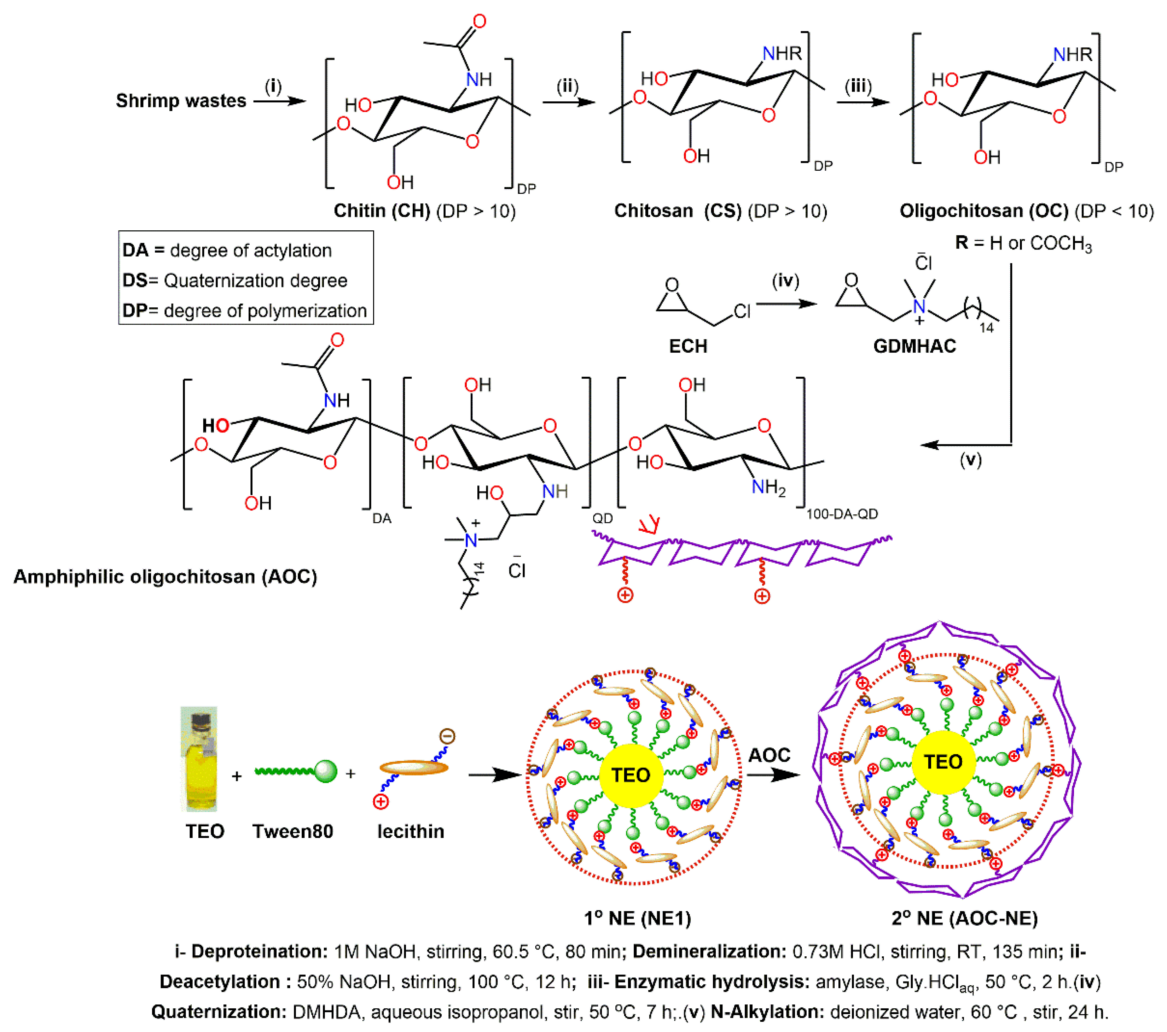
Schematic diagram for preparation of oligochitosan (OC), glycidyl *N,N*-dimethylhexadecyl ammonium chloride (GDMHAC), amphiphilic oligochitosan (AOC), and TEO-based NEs.

The stepwise protocol used to prepare the OC, AOC, and the primary, as well as secondary nanoemulsions, was shown in Scheme 1. Initially, chitosan was extracted from the shrimp wastes through three consecutive processes, namely, demineralization, deproteinization, and deacetylation. As previously reported by our research team [24,25], the controllable enzymatic degradation of chitosan with amylase mediated by aqueous glycinium ionic liquid ([Gly]Cl aq) led to the formation of oligochitosan (OC). 

On the other hand, the targeted quaternizing agent, glycidyl *N,N*-dimethylhexadecyl ammonium chloride (GDMHAC), was prepared by reaction of epoxychloropropane with N,N-dimethylhexadecylamine (DMHA). GDMHAC was then used for quaternization of OC via N-alkylation reaction to yield the corresponding amphiphilic oligochitosan (AOC), N-((2-hydroxy-3-*N,N*-dimethylhexadecyl ammonium)propyl)oligochitosan chloride.

The nonionic surfactant (Tween80) acts as an ideal emulsifier to create TEO-based nanoemulsions due to its physiochemical stability as it is less affected by pH and ionic strength changes as well as its non-toxicity and biocompatibility [36]. Therefore, Tween80 and the ionic emulsifier (lecithin) were chosen to formulate a TEO-based “primary” nanoemulsion core with a negatively charged outer surface. Afterward, the positively charged OC or AOC was adsorbed onto the negatively charged droplet surfaces of the “primary” emulsion by electrostatic interactions to create “secondary” nanoemulsions (see Scheme 1). To optimize the conditions for preparation the targeted secondary NEs, the effects of the Lec/OC weight ratio and quaternization degree (QD) of AOC on the stability of NEs were assessed.

### 3.3. Optimal Conditions for Secondary NEs Fabrication

#### 3.3.1. Optimum Lec/OC Ratio

The formulation of desired secondary nanoemulsions depends mainly on the electrostatic interaction between the anionic surface of lecithin (Lec) and anionic groups on the oligochitosan surface to form Lec-OC nanocomplex. Therefore, the Lec/OC concentration ratio will significantly affect the stability of these NEs. To investigate the optimum Lec/OC ratio, different nanoemulsions (OC-NE0 to OC-NF6) were prepared with oil to Tween80 weight ratio held constant at the previously reported optimum value of 1:9 (*w*/*w*) [36,37], and various Lec/OC ratios (1:0 to 2:1) (see Table S1, **ESI†**). All nanoformulations with lower OC concentrations (NE1, 0%; OC-NE1, 0.03; OC-NE2, 0.06%) exhibited low stability, where a phase separation can be seen after overnight storage. As the proportion of OC in nanoemulsions is increased (OC-NE3, OC-NE4, and OC-NE5 of Lec/OC of 4:1, 2:1, and 1:1 mass ratios, respectively), the dispersion and stability of NEs are significantly improved. This could be attributed to the protective layer formed by cationic OC that stabilizes the primary Lec-based NEs. Furthermore, the reduced OC concentration limits the surface coverage of the primary Lec-based NEs, resulting in lower stability of secondary NEs [38]. In contrast, the stability of secondary NEs has been significantly enhanced with the increasing OC ratio due to the increment of primary NEs coverage by the protective OC layer [38].

To learn more about why secondary NEs stability improves with increasing OC concentration, zeta potential (ZP) measurements were carried out, and the results are shown in Table S1 (**ESI†**). The ZP of the bar nanoemulsion (OC-NE0, without Lec and OC) was −4.12 mV, while a more negative ZP value was observed for NE1 (−25.69 mV) due to the incorporation of Lec in this nanoformulation. On the other hand, the values of ZP for nanoformulations OC-NE3, OC-NE4, and OC-NE5 were +15.81, +20.18, and +20.49, respectively. The elevation of zeta potential with an increase in OC concentration as well as its positive values is attributable to the formation of a cationic layer of OC on the surface of NEs. Interestingly, no significant change in ZP value can be seen with an increase in OC to Lec from 1:2 to 1:1 *w*/*w*. According to the high ZP values, the emulsion nanoparticles in OC-NE3 to OC-NE5 exhibited strong inter-particle electrostatic repulsion (strongest for OC-NE4 and OC-NE5), which led to suitable dispersion and great stability for these nanoemulsions. As a result, the OC concentration used in **OC-NE4** (0.25%, corresponding to a 2:1 Lec/OC) was chosen to be the optimal concentration to conduct further formulations and experiments.

#### 3.3.2. Protective Layer Effect

The electrostatic attractions between the ingredients of secondary emulsions provide the driving power for the adherence of the protective layer on the surface of the primary emulsion; thus, the components employed to produce the interfacial layer in secondary emulsions should have adequate electrical charges to form highly stable emulsions [39]. Although the use of the chitosan-lecithin system in the formulation of nanoemulsions leads to an improvement in their stability, nevertheless, its widespread application in nanoemulsion fabrication is restricted by the limited aqueous solubility of chitosan as well as its propensity to lose charge due to deprotonation at higher pH and consequently precipitate out of solution [40]. These facts motivated us to enhance chitosan solubility not only by its partial depolymerization into oligochitosan (OC) but also by its quaternization into amphiphilic oligochitosan (AOC) to impart to provide a persistent positive charge to it, independent of pH, and so improve the emulsion stability as well as its biological potentials. Moreover, the impacts of the AOC quaternization degree (QD) on the secondary NEs stability was investigated by measuring the particle size and zeta potential for different nanoemulsions (AOC-NE1 to AOC-NE5) (comprising TEO, 1%; Tween80, 9%; Lec, 0.5%; AOC, 0.25%). As shown in Table 2, the particle size of the ACO-based nanoemulsions was slightly reduced, while their ZPs were slightly raised, with an increase in the QD of AOC. Higher QD AOC nanoparticles produced NE with smaller nanoparticle sizes because of the greater electrostatic contact between the AOC layer and anionic Lec layer.

According to findings in Table 2, AOC-NE3 with a 39.3% QD was identified as the optimal nanoformulation for further experiments. Our results and hypotheses are generally consistent with the previous study [29].

### 3.4. Storage Stability of New NEs

**Figure 1:**
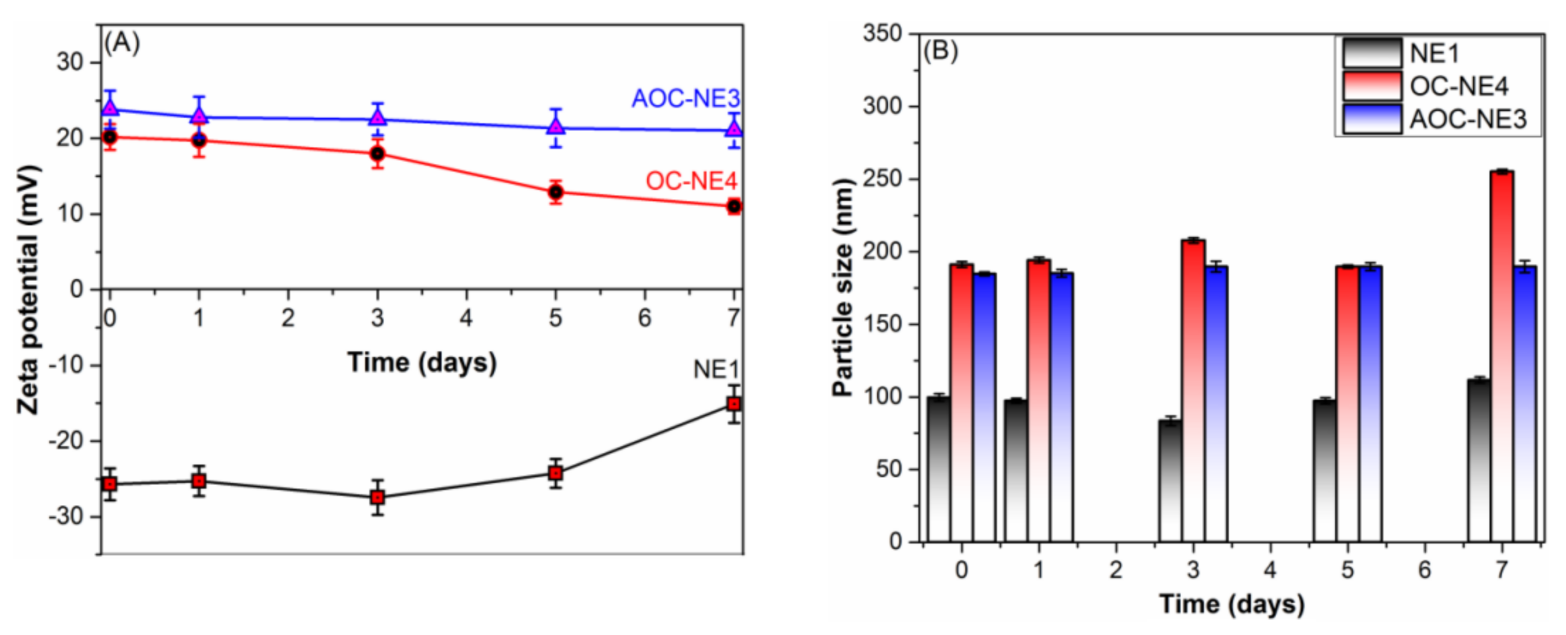
The effect of OC and AOC protective layers on the stability of their respective NEs (**A**) zeta potential (mV) and (**B**) particle size during a week of storage at room temperature, compared with bare NE (NE1).

The stability of the new NEs (**NE1**, **OC-NE4**, and **AOC-NE3**) was investigated by monitoring the changes in zeta potential and particle size of the stored NEs for a week at definite time intervals. As shown in Figure 1, in the case of bar primary nanoemulsion (NE1), the zeta potential shifted from −25.69 mV to −15.10 mV, whereas the diameter of emulsion nanoparticles increased from 99.72 nm to 111.67 nm, accompanied by an increase in the PDI (from 0.29 to 0.61). This increase in the nanoparticle size could be attributed to the Ostwald ripening of emulsion droplets [37].

On the other hand, the OC-NE4 exhibited higher stability as compared to that of NE1, as revealed by the slight fluctuations in the nanoparticle size, which remained almost constant for 5 days (from 191.27 nm to 189.77 nm) (Figure 1B); however, OC-NE4 began to show phase separation after 7 days (the reason for the abrupt increase in the nanoparticle size). In contrast, AOC-NE3 is the most stable nanoemulsion and did not show phase separation under any storage conditions, as evident by the very slight changes in the zeta potential (Figure 1A) and particle size (Figure 1B) values of the AOC-NE3 during storage. Furthermore, the PDI values remained within the desirable range (<0.3) characteristic for uniform monodisperse systems [41].

### 3.5. Physicochemical and Morphological Characterization of NEs

#### 3.5.1. Infrared Spectral Analysis

**Figure 2:**
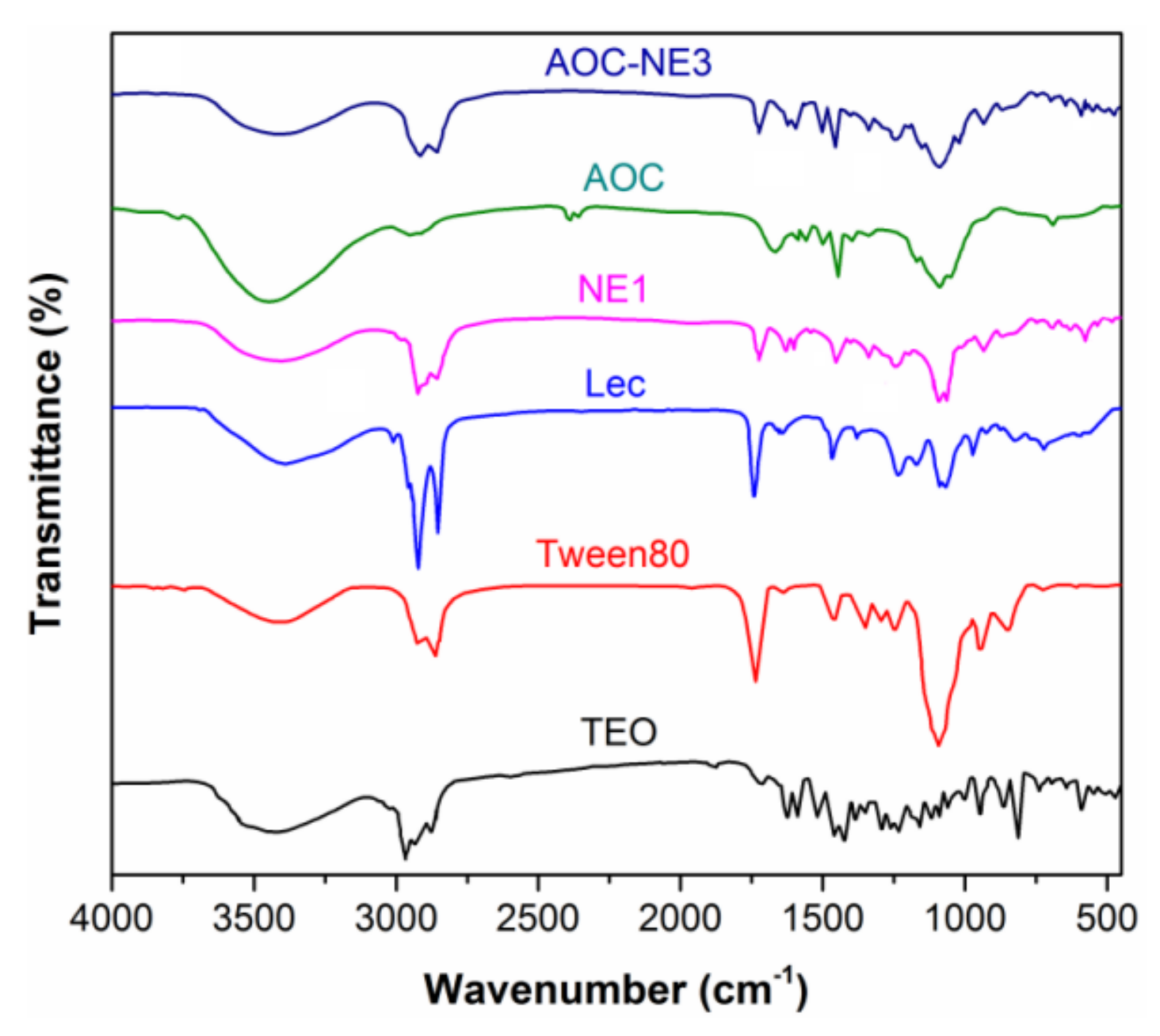
FTIR spectra of the extracted TEO, Tween80, lecithin, primary nanoemulsion (NE1), amphiphilic oligochitosan (AOC), and its secondary nanoemulsion (AOC-NE3).

The FTIR spectra of the AOC-NE3 ingredients (AOC, Lec, Tween80, and TOE) (Figure 2) were studied to confirm the successful formation of the Lec-based primary NE as well as to study the interaction between Lec and AOC to form an AOC protective layer in the secondary NE (AOC-NE3). Several IR peaks can be observed in the FTIR spectrum of TEO, of which six could be used as spectral markers for the main functional groups in oil ingredients. The broad absorption band at 3459 cm^−1^ is related to the stretching vibration of alcoholic and phenolic H-bonded O–H groups of the two main bioactive compounds (thymol, carvacrol, and carotol). The strong IR peaks observed at 2949, 1931, and 2881 cm^−1^ are assignable to the asymmetrical and symmetrical C–H stretching of methyl and isopropyl substituents on the phenyl ring, which could be related to *p*-cymene, thymol, carvacrol, and carotol [36]. The two intense stretches in the range of 1630–1584 cm^−1^ are assigned to the C=C–C vibration of aromatic rings. In addition, two sets of IR peaks can be observed in the spectral regions 1230–945 cm^−1^ and 859–809 cm^−1^ due to the aromatic C–H bending in-plane and out-of-plane, respectively. Noteworthy, the emergence of IR bands in the spectral regions 1339–1256 cm^−1^, 741–589 cm^−1^, and 1156–1058 cm^−1^, which are distinctive of the bending of the O–H bending in-plane, out-of-plane, and C–OH stretching vibrations, respectively, confirming the presence of the most bioactive component (thymol) in oil [36]. The spectrum of the Tween80 biosurfactant shows four main IR peaks at 3421, 3023, 2932, and 2864 cm^−1^, corresponding to the stretching vibrations of O-H alcoholic, C–H olefinic, C–H methyleneic, and methyl groups, respectively [42]. The presence of the carbonyl group (C=O) of the ester fragment is evident by the emergence of an intense stretch at 1736 cm^−1^. The FTIR spectrum of the ionic emulsifier “Lec” displayed IR bands at 2931, 2849, and 1735 cm^−1^ due to the C–H and C=O stretching vibration of the methylene/methyl and the ester groups, respectively. The weak IR peak observed at 1547 cm^−1^ could be attributed to the (CH_3_)_3_N^+^– stretching vibration. As indicative of the presence of phosphate linkage characteristic of the phospholipid “Lec”, is the emergence of two IR peaks at 1228 and 1063 cm^−1^, distinctive of P=O and P–O–C groups, respectively [43].

As for the FTIR spectrum of NE1 (TEO-Tween80-Lec), it can be seen that the main vibration bands of TEO (1615 cm^−1^, C=C–C; 1329 cm^−1^, (CH_3_)_3_N^+^–; phenolic O–H), Tween80 (1720, C=O ester), and Lec (1720 cm^−1^, C=O ester; 1547 cm^−1^, C=C–C; 1238 cm^−1^, P=O; 1073 cm^−1^, P–O–C) merged in this spectrum with slight shifts in their positions due to mutual interactions, which is indicative of the successful formulation of this nanoemulsion. On the other hand, the FTIR spectrum of AOC showed a broad absorption band at 3451 cm^−1^, corresponding to the stretching vibration of the O-H and N-H groups. The IR peaks distinctive of the amide I (C=O stretching) and the amide II (N-H bending) could be seen at 1642 and 1591 cm^−1^, respectively [44]. A set of IR bands observed in the range of 1155–890 cm^−1^ is related to the vibrations of the glycosidic bond (C–O–C) and the C–O group of the chitosan skeleton. In addition, the stretches observed at 2907, 2851, and 1542 cm^−1^ are assigned to the C-H stretching vibrations of the methylene, methyl, and R_4_N^+^ groups of GDMHAC. The AOC-NE3 spectrum confirms its successful formation as revealed by the emergence of IR peaks characteristic of the major functional groups of TEO, Tween80, Lec, and AOC with remarkable changes in their positions and/or intensities due to the electrostatic interactions between the quaternized amino groups of AOC and the phosphate groups of Lec.

#### 3.5.2. Scanning Electron Microscopy (SEM) Analysis

**Figure 3:**
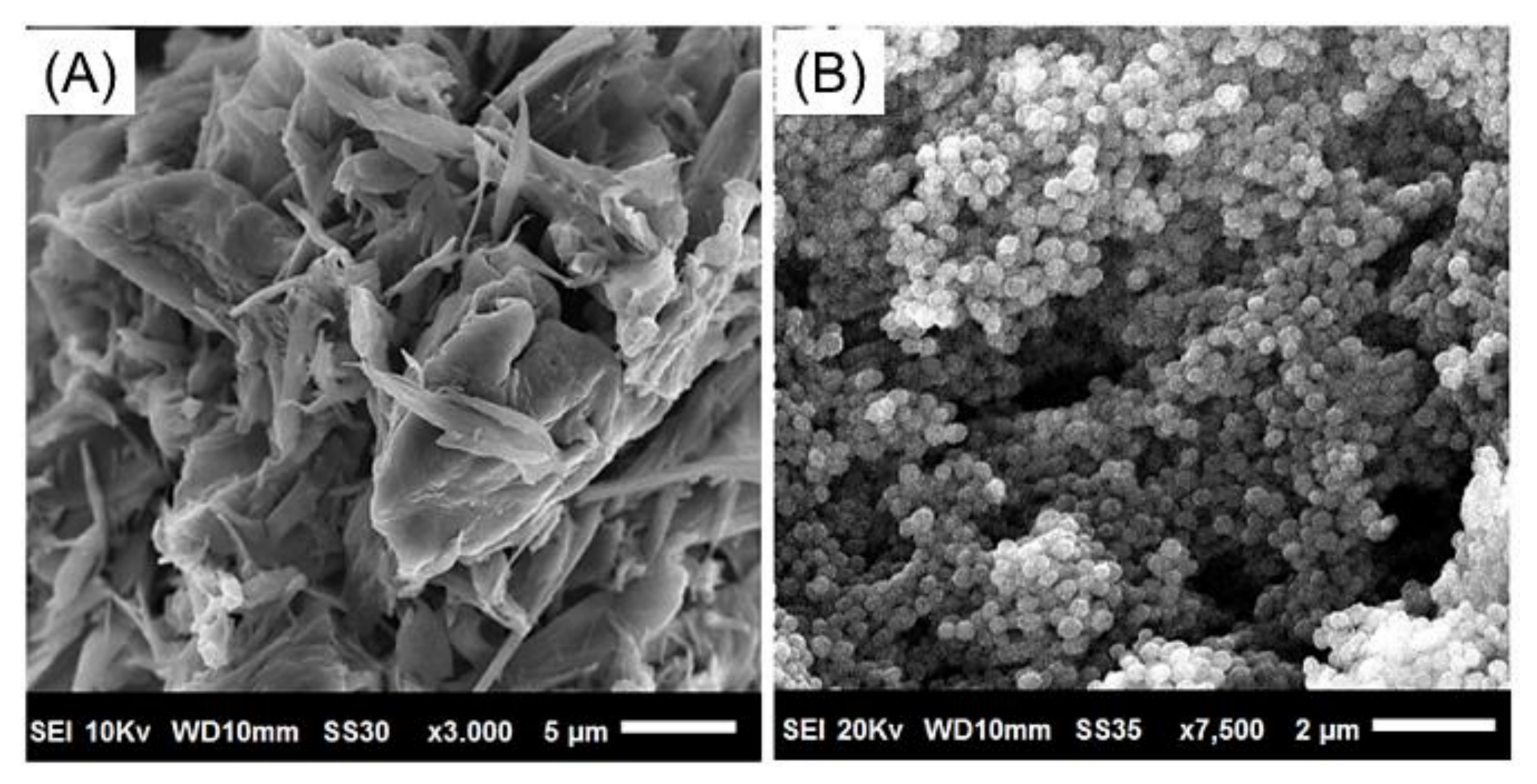
SEM micrographs of (**A**) AOC and (**B**) its secondary nanoemulsion (AOC-NE3).

The morphology of AOC and AOC-NE3 were scanned using the SEM. It is noticeable that there is a large discrepancy between the SEM micrographs of AOC and AOC-NE3 (Figure 3). As can be seen in Figure 3A, the surface of AOC exhibited interconnected flake-like shapes. On the other hand, the SEM micrograph of AOC-NE3 (Figure 3B) showed spherical thyme nanodroplets with an average particle size of ~190 nm, consistent with the mean size value obtained by dynamic light scattering (DLS). The spherical shape of the thyme droplets in the as-prepared nanoemulsions could be attributed to their low surface energy, which inhibited the rate of undesirable phenomena that commonly occurred in the prepared nanoemulsions, such as creaming and flocculation [36]. In addition, the high values of zeta potential of the as-fabricated nanoemulsions (OC-NE4, AOC-NE3) (Table 2) reflect the strong inter-repulsion between the formed nanodroplets in the NEs, which enables nanodroplets to sustain their spherical shape.

#### 3.5.3. Encapsulation Efficiency and Drug Loading

The calculated EE and OL for OC-NE4/AOC-NE3 were 78.6/91.5% and 1.8/3.1%, respectively, indicating superior entrapment and encapsulation performance of the Lec-AOC system as compared to the Lec-OC system. The extremely high EE could be attributed to the excellent emulsifying properties of lecithin, which enable it to greatly emulsify the oil, as well as the presence of a permanent cationic charge on the AOC surface, which enables it to form flexible and efficient interaction with the negatively charged Lec. Similar findings and hypotheses were reported by Luesakul et al. [29]. In addition, the Lec-AOC complex presents better interfacial properties than its native components. Moreover, the loss in TEO (8.5%) might be due to the evaporation of volatile ingredients in oil during the nanoemulsion preparation.

#### 3.5.4. The pH Sensitivity

As aforementioned, the poor solubility of chitosan at physiological pH (pKa 6.3) is the main obstacle to its widespread pharmacological applications. Therefore, AOC was prepared to obtain a low-molecular-weight biopolymer with a sustainable positive surface and excellent aqueous solubility at physiological pH. It is well known that the protective layer’s (AOC) high charge density increases its proclivity for adherence to the primary emulsion layer. As a result, the influence of pH on the charge density (zeta potential) and particle size of OC-NE4 and AOC-NE3 was examined at pH values of 4.5 and 7.2. At acidic pH (4.5), both NEs (OC-NE4 and AOC-NE3) displayed high positive charge density with zeta potential values of about 22 and 25 mV, respectively. Furthermore, the particle size for OC-NE4 and AOC-NE3 was ~190 and ~180 nm, respectively. As the pH increased to 7.2, the degree of protonation of free NH_2_ groups of OC and AOC decreased, resulting in a lowering of the positive charge density on OC and AOC; therefore, the values of zeta potential for OC-NE4 and AOC-NE3 were decreased by 7 mV and 8 mV, respectively. As a result, the mutual electrostatic interactions between the amino group of the protective layer (OC or AOC) and the phosphate group of Lec greatly decreased, which led to a dramatic increase in the particle size of OC-NE4 (890 nm), while the particle size of AOC-NE3 was increased to 340 nm. This could be because the interfacial layer becomes less compact, causing the formation of large pores and voids in the secondary NEs matrix, allowing the entrapment of H_2_O molecules into emulsion nanodroplets and consequently their swelling to a great extent. In summary, acidic conditions are preferred to prepare the desired NEs with optimal characteristics. Moreover, a pH range of 4–6 has been suggested for topical therapy emulsions, which is near to the skin’s natural pH level [45]. 

### 3.6. Pharmacological Performance

#### 3.6.1. Anti-Inflammatory Activity

**Figure 4:**
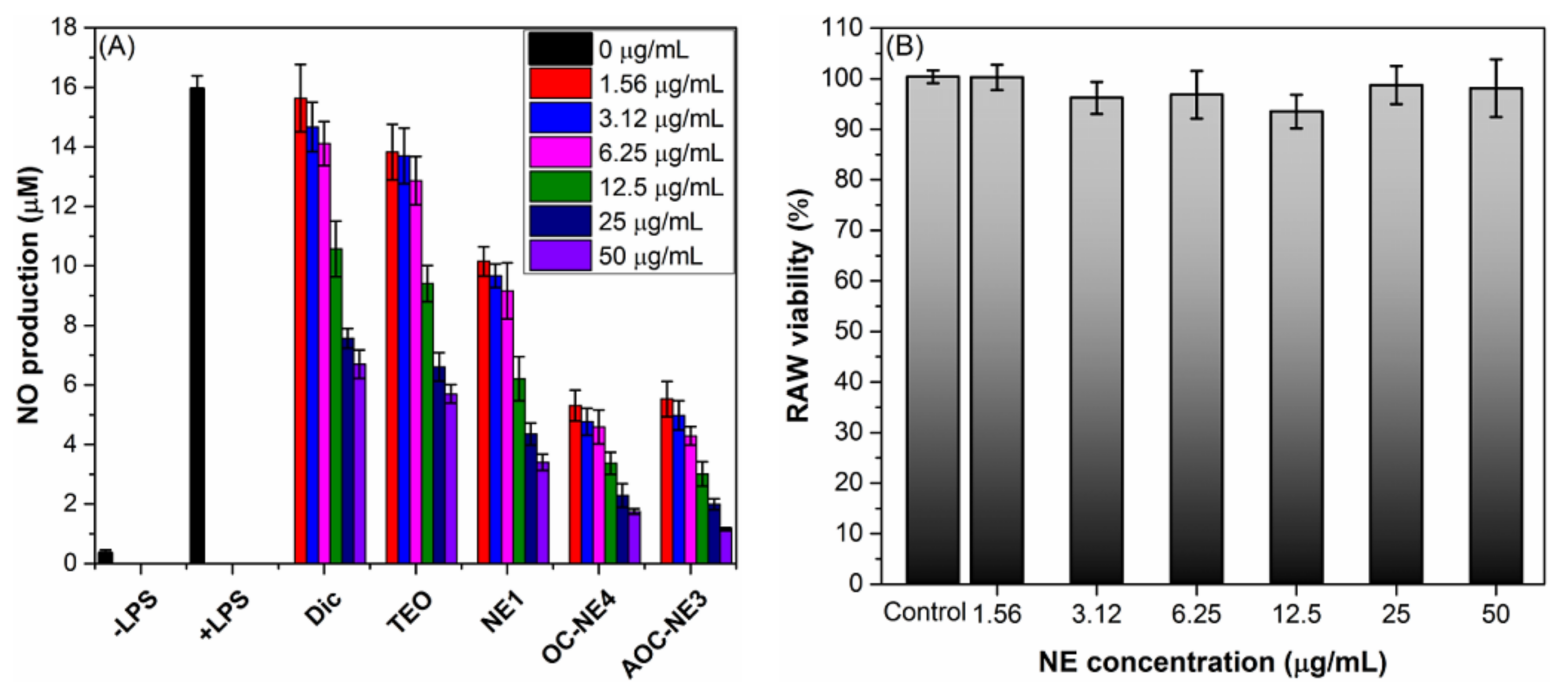
(**A**) Effect of Dic, TEO, and TEO-based NEs (NE1, OC-NE4, and AOC-NE3) at progressive concentrations of (0–50 μg/mL) on the nitric oxide formation in LPS-induced RAW 264.7 cells. (**B**) Changes in the RAW 264.7 cells viability after treatment by AOC-NE3 for 48 h.

The production of nitric oxide (NO), an inflammatory mediator, is induced by the enzymes and pro-inflammatory cytokines such as TNF-α, IFN-γ, and IL-1β [46]. Therefore, NO production could be used to estimate the inflammation extent [46]. Thyme extract has been shown to have anti-inflammatory properties by lowering the synthesis and gene expression of the pro-inflammatory cytokines TNF-, IL-1B, and IL-6 while raising these indicators for the cytokines IL-1 and IL-2 (IL-10) [47,48]. The anti-inflammatory capability of TEO motivated us to investigate the impact of NEs formulation on the anti-inflammatory properties of thyme essential oil (TEO) in LPS-induced macrophages by measuring the nitric oxide formation using Griess reagent. Diclofenac (Dic), a nonsteroidal anti-inflammatory drug (NSAID), was used as a positive control. As shown in Figure 4A, after 48 h of incubation, the RAW 264.7 cells produced very small amounts of “NO” (0.35 μM) without LPS activation, while “NO” production was significantly increased to 15.94 μM in LPS-activated cells. All the tested samples have the capacity to reduce “NO” production, and their performance depends on the ingredients and dose of the sample. Generally, the “NO” production inhibitory effect and, subsequently, the anti-inflammatory activity of AOC-NE3 > OC-NE4 > NE1 > TEO > Dic. For instance, treatment of RAW 264.7 cells with 25 μg/mL of TEO diminished the LPS-induced NO production to 5.59 μM (64.9% inhibition), while Dic showed a lower inhibitory effect toward “NO” production with a 52.3% inhibition. On the other hand, treatment of RAW 264.7 cells with TEO-based NEs (NE1, OC-NE4, and AOC-NE3) under the same conditions inhibited the “NO” production by 72.7, 85.6, and 87.5%, respectively, which is significantly higher than the *Thymus* extract (*p* < 0.05). Thus, the incorporation of *Thymus* extract (TEO) into NEs (NE1, OC-NE4, and AOC-NE3) has greatly improved its anti-inflammatory activity in LPS-induced macrophages. Interestingly, the positively charged NEs (OC-NE4 and AOC-NE3) exhibited significantly (*p* < 0.05) higher activity than the negatively charged ones (NE1). Noteworthy, AOC-NE3 showed a slightly better anti-inflammatory effect than OC-NE4. This could be due to the multiple anti-inflammatory effects of TEO and multifunctional components of AOC (amphiphilic segment and oligochitosan) because OC itself could inhibit various pro-inflammatory cytokines [49]. 

To validate that the NO suppressive action of the most anti-inflammatory NE (AOC-NE3) was not due to its cytotoxicity, the cytotoxic effect of AOC-NE3 on RAW 264.7 cells treated with serial concentrations of AOC-NE3 was assessed, and their viability was estimated relative to a control (cell viability 100%) by the MTT assay. As shown in Figure 4B, there was no significant change in the viability of macrophages upon treatment with AOC-NE3, where the survival of RAW 264.7 cells treated with 50 μg/mL of AOC-NE3 for 48 h was still > 98%. These findings validated that AOC-NE3 is not toxic for RAW 264.7 cells and the NO suppressive action of it at the highest dose was not due to its cytotoxicity impact.

#### 3.6.2. Ex Vivo Skin Permeability Study and Release Kinetics

**Figure 5:**
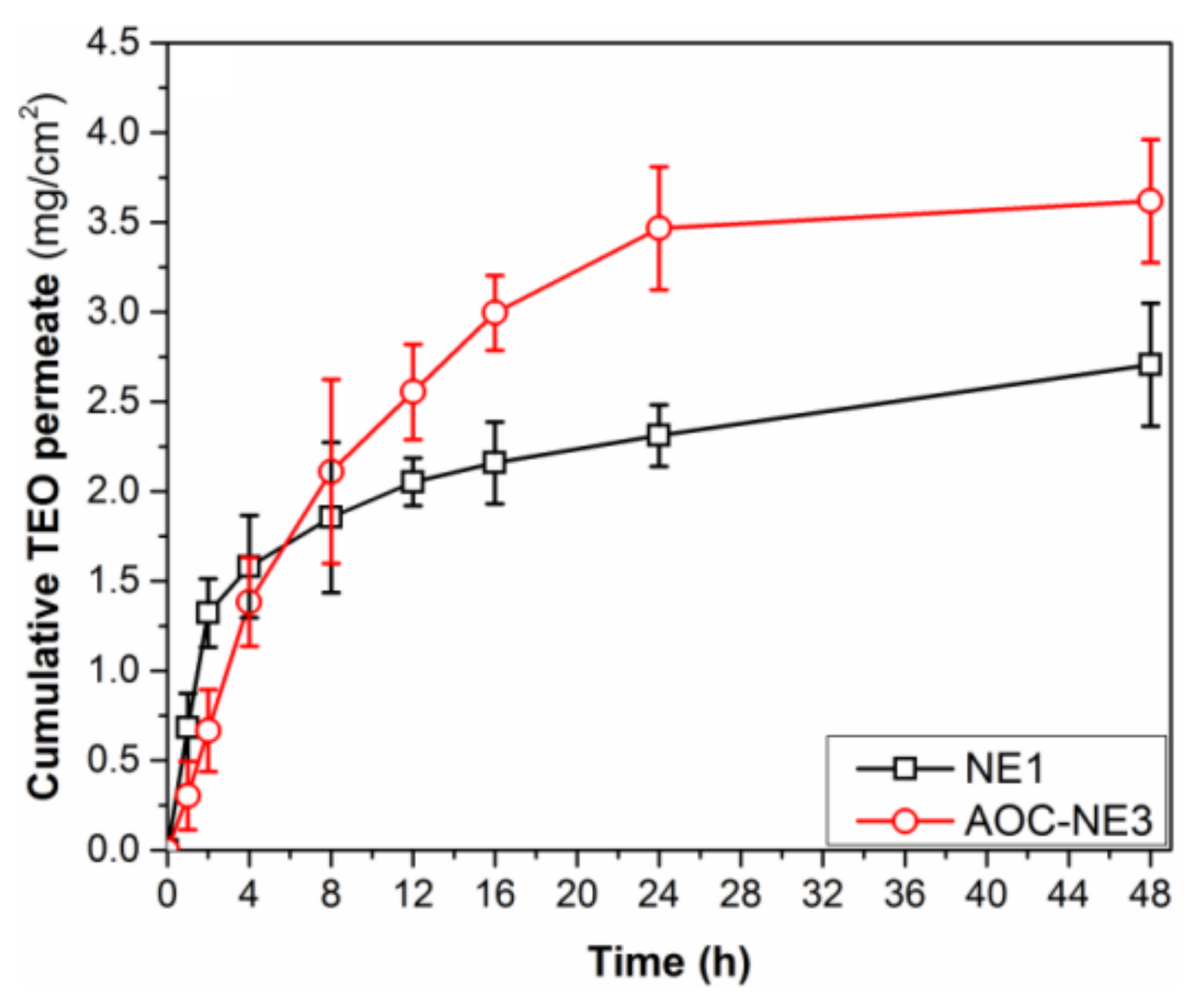
Skin permeability profile of TEO-based nanoformulations (NE1 and AOC-NE3) through a rat skin for 24 h.

The *Thymus vulgaris* extract has a poor capacity to penetrate the skin [50], so the use of NEs could offer a promising strategy to overcome the difficulties related to the low skin permeability of TEO for its topical applications. Therefore, the ability of the most potent anti-inflammatory NE (AOC-NE3) to penetrate the skin barrier was ex vivo studied, in comparison to the negatively charged NE (NE1), using rat skin as a natural membrane using Franz diffusion cell. The content of TEO in the skin leachables was determined spectrophotometrically (Figure S2, **ESI†)**. The spectra of the native nanoemulsion and its skin permeate are virtually identical, with minor differences in peak intensities due to the variation in oil content in NE and permeate, as well as the appearance of a very weak wide peak around 450 nm. These data indicated that skin leachables had no effect on the NE permeation testing [51]. The correlation between the cumulative amount of TEO-based NEs that permeate through the unit area of rat skin and time is depicted in Figure 5. In the first 2 h, the uncoated NE (NE1) showed significantly (*p* < 0.05) higher skin permeability (1.32 mg/cm^2^ permeation) than that of the coated one (AOC-NE3) (0.66 mg/cm^2^ permeation). Surprisingly, the results of permeation within the first 2 h contradict prior claims in several publications that promise enhanced penetration with positive charge NEs. This could be related to the smaller size of NE1 (~100 nm) as compared to AOC-NE3 (~185 nm), which has a significant effect on the permeability of bioactive components through the skin. NE particle size greatly affects skin bioactive delivery. Previous studies tested 80, 200, and 500 nm NE skin penetration. Small NEs, such as 80 nm, can breach the viable epidermis and fill hair follicles, but larger NEs, such as 500 nm, can only travel along hair follicles. The 200 nm NEs have modest transdermal delivery effects [29]. On the other hand, after 4 h, the permeation style for AOC-NE3 was surpassed that of NE1 until reaching a plateau at 24 h. By the end of the first day, the skin permeation for AOC-NE3 was 3.47 mg/cm^2^, which is significantly (*p* < 0.05) higher than that for NE1, 2.31 mg/cm^2^. These findings revealed the importance of the positive charges on the surface of AOC-NE3 in enhancing its skin permeability by ~1.5-fold in comparison to the negatively charged NE. The permeability parameters, including steady-state transdermal flux (*J*_ss_, mg cm^–2^ h^–1^), permeability coefficient (*K*_p_, cm h^–1^), and enhancement ratio (*E*_r_), were calculated, and their mean values are presented in Table 3: 

As shown in Table 3, a significant increase in the values of *J*_ss_, *K*_p_, and *E*_r_ was observed for NE formulations NE1 and AOC-NE3 as compared to TEO (*p* < 0.05). Moreover, AOC-NE3 exhibited higher permeability parameters (*J*_ss_, 0.2313 mg cm^–2^ h^–1^; *K*_p_, 4.61 × 10^−3^ cm h^–1^; *E*_r_, 4.72), than that of NE1 (*J*_ss_, 0.0763 mg cm^–2^ h^–1^; *K*_p_, 0.92 × 10^−3^ cm h^–1^; *E*_r_, 1.56). This higher skin permeability of AOC-NE3 could be attributed to the high positive charge density of its nanodroplets (ZP = +23.82 ± 0.55 mV), which enables it to be strongly attached to the negatively charged cell membrane of rat skin, and, consequently, provide enough time for the nanodroplets to penetrate the skin. In contrast, the negative charge of NE1 (ZP = −25.71 ± 0.95 mV) induces repelling from the negatively charged rat skin cell membrane [32].

### 3.7. In Vitro TEO Release Kinetics 

According to the literature, a two-step process was used to determine how TEO would release from the polymer network. In the first stage, oil molecules infiltrate the polymer matrix, causing swelling and weakening of the polymeric network structure. Afterward, the essential oil molecules diffuse from the film’s inside to the simulant solution (PBS) until thermodynamic equilibrium is reached [52]. To investigate the release characteristics of the TEO-based NEs (NE1 and AOC-NE3), the results obtained from the in vitro release experiments (Section 2.4) were fitted to different kinetic model equations. The values of coefficient of correlation (*R*^2^) were used to find the best fitted kinetic model. As shown in Table 3, the Higuchi model for NE1 and AOC-NE3 was shown to be the most appropriate one to describe the TEO release kinetic, demonstrating that NE1 and AOC-NE3 have been shown to be permeable by diffusion. These findings agree well with the previously reported study for the release of TEO from the TEEO nanoemulsion (TEON) incorporated polyelectrolyte gellan gum (GG)-chitosan (CS) multilayer films [33]. 

#### 3.7.1. Validation of UV-Vis Spectrophotometric Method for TEO Estimation

The TEO nanoemulsion’s absorbance was linear between 1.25 and 40.0 mg/mL with calibration curve equation 0.0155× + 0.5319 and regression coefficient of (*R*^2^) = 0.9968 (see Figure S3, **ESI†**). The TEO-NE spectrophotometric estimation curve’s suitable linearity confirms the accuracy of this method for TEO estimation. The calculated QL and DL values were 0.469 and 0.156 mg/mL, respectively. 

#### 3.7.2. Anticancer Activity

Motivated by the high transdermal permeability and anti-inflammatory potentials of the new TEO-based nanoemulsions, as well as the moderate anticancer activity of native TEO [17,53,54], it is interesting to study the effects of NEs formulation on the anti-skin cancer activity of thyme extract (TEO). To that end, the antiproliferative activities of the native *Thymus* extract (TEO) and new TEO-based NEs (NE1, OC-NE4, and AOC-NE3) with serial concentrations (1.56–100 μg/mL) were investigated against melanoma cell lines (A-375) and normal human skin fibroblast cells (HSF) using the MTT assay. According to the obtained results (Figure S2, **ESI†**), the incorporation of TEO into nanoemulsions significantly enhanced its anti-melanoma performance in comparison to the native TEO, as revealed by the great reduction in the A-375 cellular viability upon treatment with these NEs. Furthermore, the cytotoxic performance was dose-dependent and nanoemulsion ingredients-dependent. It can be seen in Figure S3 (**ESI†**) that the antiproliferative activity increased with increasing the sample dose. Moreover, the positively charged NEs (OC-NE4 and AOC-NE3) exhibited significantly (*p* < 0.05) higher activity than the negatively charged ones (NE1). This may be because of the positive charge on their surfaces, which enables them to strongly adhere to the negatively charged phosphatidylserine (PS) expressed on the melanoma surface [55] and consequently provide enough time to penetrate cancer cells and induce their apoptosis.

As shown in Table 4, TEO exhibited very low cytotoxicity toward A-375 cells, recoding an IC_50_ of (76.13 ± 2.35) μg/mL. In contrast, TEO-containing NEs (NE1, OS-NE4, and AOC-NE3) showed more significantly enhanced cytotoxicity (*p* < 0.05) as compared to the native TEO, 3- to 5-times lowering in the values of IC_50_. Among all the NEs, the AOC-coated NE (AOC-NE3) showed the most potent antiproliferative activity against the A-375 cancer cells, with an IC_50_ value of 14.38 ± 1.05 μg/mL. This could be due to its highest surface positive charge density (ZP = +23.82 ± 0.55 mV), which makes it more effective at penetrating cancer cells owing to its strong interactions with the negatively charged outer surface of cancer cells.

The effects of progressive concentrations (1.56–200 μg/mL) of these NEs on the growth of normal HSF cells were also tested in order to investigate the selectivity of the freshly produced NEs for targeting cancer cells over normal cells. As shown in Figure S3 (**ESI†**), all tested formulations exhibited lower antiproliferative effects on the HSF cells as compared to their antiproliferative effects on the melanoma cells (A-375) under the same testing conditions. Meanwhile, the values IC_50_ were ranged from 104.29 ± 3.11 μg/mL (for native TEO) to 72.19 ± 2.39 μg/mL (for AOC-NE3), as represented in Table 4. It is also possible to determine the selectivity of new nanoformulations for cancer cells (A-375) over normal cells (HSF) by calculating the selectivity index (SI) for them using the following equation (Equation (10)) [56].

(10) $$\mathrm{SI}=\frac{{\mathrm{IC}}_{50}\;\mathrm{for}\;\mathrm{tested}\;\mathrm{formulation}\;\mathrm{against}\;\mathrm{HSF}\;\mathrm{cells}}{{\mathrm{IC}}_{50}\;\mathrm{for}\;\mathrm{tested}\;\mathrm{formulation}\;\mathrm{against}\;A-375\;\mathrm{cells}}$$

Interestingly that the most potent anti-melanoma agent (AOC-NE3) showed the highest selectivity level for attacking A-375 cells over normal cells (HSF) (SI = 5.02). Thus, AOC-NE3 could act as a promising and safe anti-melanoma candidate. Overall, results have evident that NEs formulation could enhance the *Thymus* extract’s anticancer activity by enhancing the *Thymus* extract’s anti-inflammatory properties. Nevertheless, the MTT assay is employed as a first step in the search for anticancer candidates. Further in vivo and in vitro experiments are necessary to establish the anticancer efficacy of *Thymus* extract and its NEs. This will be the subject of our future studies.

## 4. Conclusions

This study succeeded in extracting thyme essential oil (TEO) and oligochitosan (OC) from *Thymus vulgaris* L. (thyme) and shrimp wastes, respectively, using facile protocols. Moreover, quaternization of OC was performed using glycidyl ammonium chloride (GAC) to yield the corresponding amphiphilic oligochitosan (AOC). On the other hand, a simple and low-energy protocol was successfully applied to prepare a primary nanoemulsion (NE1) incorporating TEO, lecithin, and Tween80. The OC and AOC were then used to formulate coating protective layers on the NE1 surface, targeting the preparation of highly stable secondary nanoemulsions (OC-NE4 and AOC-NE3) for potential transdermal applications. The new nanoformulations were structurally and morphologically characterized using spectral, microscopic, and electrical measurements. After coating NE1 with a protective layer (OC or AOC), the particle size of NE was increased, whereas the zeta potential was converted from a negative to a positive value. According to the findings of the storage stability experiment, the protective layer has greatly enhanced the stability of the as-fabricated NEs (OC-NE4 and AOC-NE3). Among all the nanoformulations, the AOC-coated NE is the most stable one, where it could be stored for up to a week without obvious changes in its respective properties (particle size, PDI, and zeta potential). In addition, the protective layer has significantly increased transdermal delivery of the secondary NEs. Consequently, AOC-NE3 had a transdermal delivery 1.5 times greater than primary nanoemulsion (NE1). The in vitro kinetic release experiment proposed that the Higuchi model is the best fit style for releasing TEO from the new nanoemulsions by a diffusion mechanism. Interestingly, in LPS-induced macrophages (RAW 264.7), the secondary nanoemulsions (OC-NE4 and AOC-NE3) showed much stronger anti-inflammatory capabilities than the bar primary nanoemulsion and native TEO. The cytotoxicity assay revealed that all of the nanoemulsions can induce a dose- and structure-dependent decrease in the proliferation of melanoma cells (A-375), with AOC-NE3 having the greatest efficacy (a 5-fold lower IC_50_ value when compared to TEO). In contrast, AOC-NE3 showed low toxicity toward normal (HSF) cells. Consequently, AOC-NE3 exhibited excellent selectivity for melanoma cells over the healthy cells, as revealed by its selectivity index (SI = 5.02). According to these findings, the AOC-coated nanoemulsion technology can be employed to deliver lipophilic active components transdermally.

## Figures and Tables

**Table 1:** Chemical ingredients (listed according to concentration) of *Thymus* extract (TEO) using GC-MS.

No.	Compound	RT (min)	Yield (%)	No.	Compound	RT (min)	Yield (%)
**1**	Germacrene D	12.43	10.1	**26**	7-Hydroxyfarnesen	18.09	0.99
**2**	Thymol	9.21	8.32	**27**	α-Gurjunen	10.85	0.88
**3**	Carotol	16.57	7.6	**28**	1H-Cyclopropa[a]naphthalene	18.23	0.85
**4**	Carvacrol	9.51	6.96	**29**	Isolongifolene oxide	19.72	0.83
**5**	α-Acorenol	13.3	6.25	**30**	6-epi-shyobunol	16.74	0.81
**6**	D-Germacren-4-ol	14.31	5.61	**31**	Aristol-1(10)-en-9-yl Isovalerate	18.28	0.75
**7**	(-)-Globulol	13.39	4.75	**32**	8,9-dehydro-Cycloisolongifolene	18.32	0.75
**8**	Cubebanol	12.67	4.47	**33**	Cubebanol	14.48	0.74
**9**	Humulene	11.79	4.12	**34**	Nonadecatriene-5,14-diol	18.75	0.71
**10**	α-Copaen	10.19	3.86	**35**	6-epi-shyobunol	13.67	0.65
**11**	Isocaryophillene	11.07	3.58	**36**	8,9-dehydro-Neoisolongifolene	18.25	0.65
**12**	2,4-Patchouladiene	18.19	3.09	**37**	*p*-Mentha-1-ene-6-one	9.59	0.64
**13**	Ledene oxide-(II)	19.49	2.03	**38**	β-Selinene	12.8	0.61
**14**	(-) β-Elemene	10.56	1.85	**39**	(-)-Spathulenol	20.24	0.61
**15**	α-Cadinol	15.97	1.75	**40**	Isolongifolene oxide	20.51	0.61
**16**	α-Pinene oxide	16.91	1.69	**41**	Ledene oxide-(II)	20.39	0.59
**17**	τ-Muurolol	15.53	1.68	**42**	Thunbergol	21.43	0.59
**18**	Germacrene A	10.69	1.35	**43**	D-Guaiene	12.85	0.57
**19**	Caryophyllene oxide	18.02	1.31	**44**	Cyclooctenone dimer	18.92	0.57
**20**	Cholestan-3-ol	18.68	1.22	**45**	Alloaromadendrane-4β,10α-diol	19.86	0.57
**21**	Junenol	13.42	1.15	**46**	Ledol	16.85	0.56
**22**	Cembrene	21.13	1.13	**47**	α-Yalangene	10.32	0.55
**23**	Nerolidol-Epoxyacetate	18.56	1.05	**48**	Longifolenaldehyde	18.14	0.53
**24**	τ-Cadinol	15.79	1.04	**49**	o-Cymene	6.92	0.45
**25**	(-)-Spathulenol	19.57	1.03				

**Table 2:** Impact of OD of AOC on the stability indices of AOC-based NEs (particle size, PDI, and ZP).

NE	GDMHAC:OC	QD%	Particle Size (nm)	PDI	ZP (mV)
AOC-NE1	1.0:1.0	20.6	193.91 ± 2.12	0.23	21.83 ± 0.58
AOC-NE2	1.5:1.0	29.5	194.63 ± 3.65	0.21	24.91 ± 0.42
AOC-NE3	2.0:1.0	39.3	184.74 ± 1.27	0.19	23.82 ± 0.55
AOC-NE4	2.5:1.0	44.4	180.85 ± 1.75	0.23	23.99 ± 0.48
AOC-NE5	3.0:1.0	47.5	179.03 ± 1.98	0.22	24.78 ± 0.39

**Table 3:** Permeability parameters (*J*_ss_, *K*_p_, and *E*_r_) and correlation coefficients for the in vitro release study of TEO based on different kinetic models of NE1 and AOC-NE3 nanoformulations.

Sample	Permeate in 24 h (mg cm^–2^)	*J*_ss_ (mg cm^–2^ h^–1^)	*K*_p_ × 10^−3^ (cm h^–1^)	*E*_r_	*R* ^2^		
					Zero Order	1st Order	Higuchi
**TEO**	–	0.0491 ± 0.01	0.17 ± 0.05	–	–	–	–
**NE1**	2.31 ± 0.15	0.0763 ± 0.03	0.92 ± 0.12	1.56 ± 0.48	0.79044	0.63515	0.91442
**AOC-NE3**	3.47 ± 0.13	0.2313 ± 0.10	4.61 ± 0.83	4.72 ± 0.83	0.95937	0.78812	0.99853

**Table 4:** IC_50_ (μg/mL) and SI values for *Thymus* extract (TEO) and its NEs against melanoma (A-375) and human skin fibroblast (HSF) cells.

Cell Line	IC_50_ (μg/mL) ± SD			
	TEO	NE1	OC-NE4	AOC-NE3
**A-375**	76.13 ± 2.35	27.11 ± 1.33	18.78 ± 1.56	14.38 ± 1.05
**HSF**	104.29 ± 3.11	79.42 ± 2.87	74.86 ± 1.85	72.19 ± 2.39
**SI**	1.37	2.92	3.99	5.02

## Data Availability

Not applicable.

## Supplementary Materials

The following supporting information can be downloaded at: www.mdpi.com/article/10.3390/pharmaceutics14071350/s1. Figure S1: GC-MS chromatogram of the extracted TEO, Figure S2: UV-vis spectra of excipients (native) and the skin leachables TEO-nanoemulsion, Figure S3: Calibration curve of TEO-nanoemulsion; Table S1: Compositions and values of zeta potential for the OC-based NEs. Reference [57] are cited in the supplementary materials.

## Abbreviations

**AOC**, amphiphilic oligochitosan; **AOC-NE,** amphiphilic oligochitosan-coated nanoemulsion; **EE**, Encapsulation efficiency; **EOs**, essential oils; **Lec**, lecithin; **NEs**, nanoemulsions; **NEDSs**, nanoemulsion delivery systems; **OC-NE,** oligochitosan-coated nanoemulsion; **OL**, oil loading; **RS,** rat skin; **SC**, stratum corneum; **TEO**, thyme essential oil.
