# Supplementary material for: Upgrading the Transdermal Biomedical Capabilities of Thyme Essential Oil Nanoemulsions Using Amphiphilic Oligochitosan Vehicles
Source: Pharmaceutics. 2022 Jun 25;14(7):1350. doi: 10.3390/pharmaceutics14071350 (PMC9317589; doi:10.3390/pharmaceutics14071350)
Supplement: Supplementary file 1 [file pharmaceutics-14-01350-s001.zip › pharmaceutics-1745025-supplementary.pdf]

Supplementary information

Upgrading the transdermal biomedical capabilities of Thyme essential oil nanoemulsions using amphiphilic oligochitosan vehicles

Ali M. Nasr [a], Mohammad Y. Alfaifi [b], Ali A Shati,[b] Serag Eldin I. Elbehairi,[b,c] Reda F. M. Elshaarawy [d,e],* Islam Kamal [a]

[a] Department of Pharmaceutics, Faculty of Pharmacy, Port Said University, 42526 Port Said, Egypt.

[b] Biology Department, Faculty of Science, King Khalid University, 9004 Abha, Saudi Arabia

[c] Cell Culture Lab, Egyptian Organization for Biological Products and Vaccines (VACSERA Holding Company), Giza 12311, Egypt

[d] Department of Chemistry, Faculty of Science, Suez University, 43533 Suez, Egypt.

[e] Institut für Anorganische Chemie und Strukturchemie, Heinrich-Heine Universität Düsseldorf, Düsseldorf, Germany.

Corresponding Author; RFME
E-mail: reda.elshaarawy@suezuniv.edu.eg; reel@hhu.de

1. Materials

Chemicals were obtained from the following suppliers and used without further purification: glycine, (Sigma–Aldrich) and silver nitrate ($AgNO_3$) (GRÜSSING GmbH).

The crab shell waste used in this study was obtained from the Suez fish market which obtained the shrimp from the Suez Gulf, Suez, Egypt. The shrimp shells were washed thoroughly with water and then dried at a specialized facility by pressing with a screw press to eliminate the majority of the water, followed by heating at up to 160 °C in a fluidized bed dryer until the material had a final moisture content of less than 5 wt%. The dried shrimp shells was ground using a ball mill and sieved to give a powder with a particle size of <250 μm. Ground crab shells were further dried at 80 °C for 24 h. The chitin content of the dried crab shell was measured by the Black and Schwartz method [57]. Moreover, the moisture and $CaCO_3$ contents in shrimp shells were determined gravimetrically, while, the residual ash content was determined by mass balance. According to the results of these experiments, the composition of shrimp shells was found to be: chitin content (24%); moisture (4%); $CaCO_3$ content (25%), and residual (47%).

2. Instrumentation

Elemental analyses as performed with a Perkin–Elmer 263 elemental analyzer. FT-IR spectra were recorded on a BRUKER Tensor-37 FT-IR spectrophotometer in the range 400–4000 cm^{-1} as

KBr discs or in the 4000-550 cm^{-1} region with 2 cm^{-1} resolution with an ATR (attenuated total reflection) unit (Platinum ATR-QL, Diamond). For signal intensities the following abbreviations were used: br (broad), sh (sharp), w (weak), m (medium), s (strong), vs (very strong). The electronic absorption spectra were obtained using a 1 cm quartz cell using an Evolution™ 200 series UV-Visible spectrophotometer. Transmission electron microscopy (SEM) images were taken by (SEM-EDX, Hitachi S-7400, Hitachi, Japan).

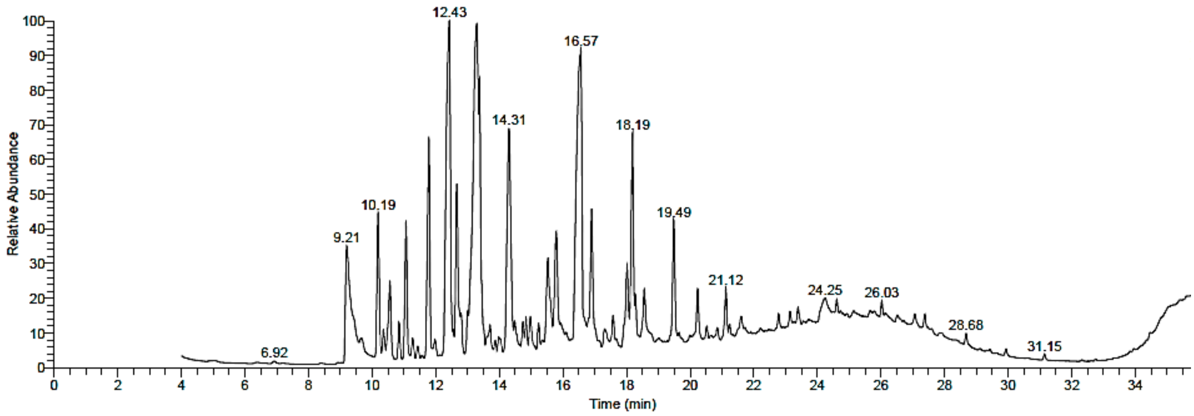

Figure S1. GC-MS chromatogram of the extracted TEO.

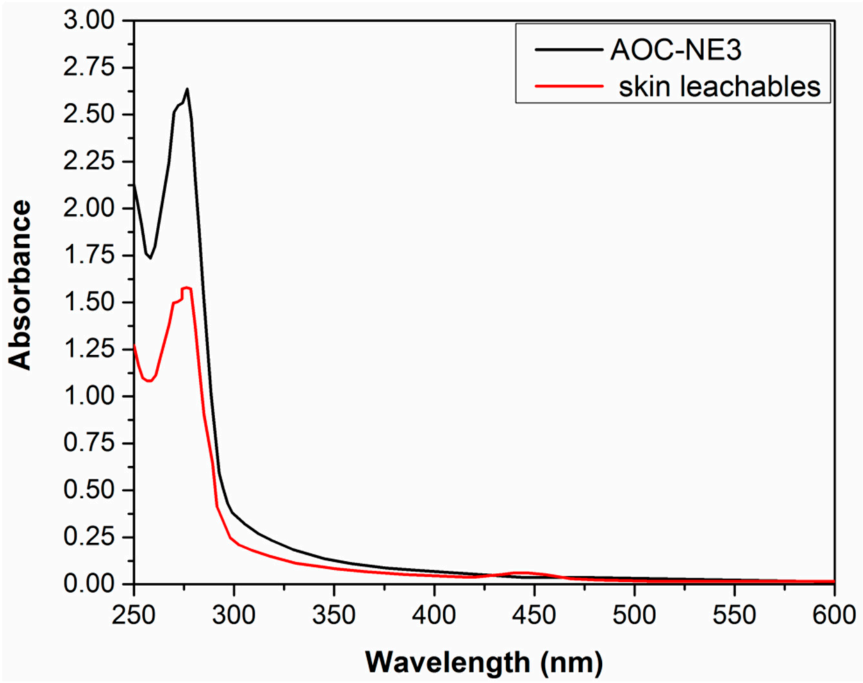

Figure S2. UV-vis spectra of excipients (native) and the skin leachables TEO-nanoemulsion.

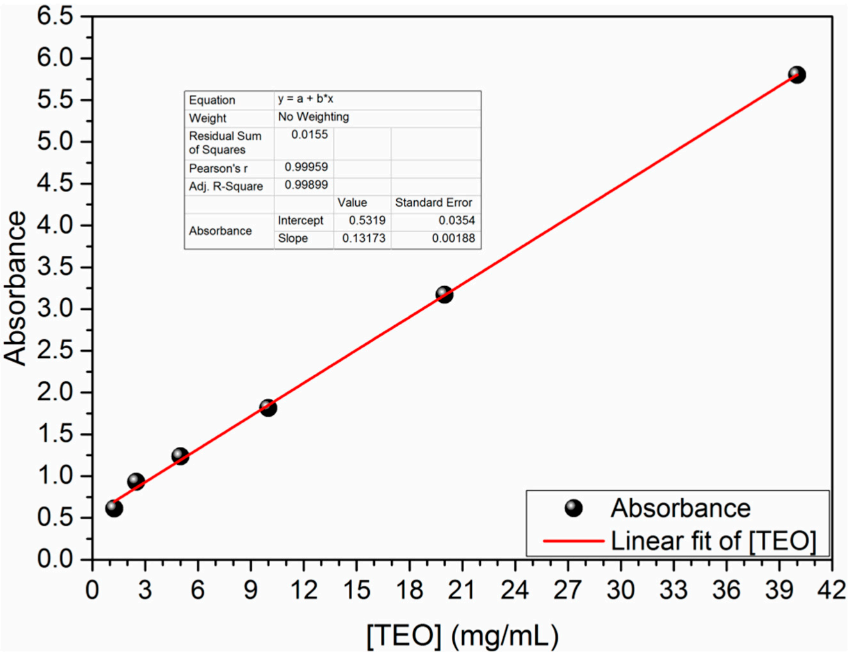

Figure S3. Calibration curve of TEO-nanoemulsion.

Table S1. Compositions and values of zeta potential for the OC-based NEs

**Formulations**	**Composition (%w/w)**				**Lec/OC**	**ZP (mV)**
	**TEO**	**Tween20**	**Lec**	**OC**		
NE0	5	20	0.0	0.0	-	−4.12
NE1	5	20	0.5	0.0	1:0	−25.69
OC-NE1	5	20	0.5	0.03	16:1	−11.69
OC-NE2	5	20	0.5	0.06	8:1	+2.69
OC-NE3	5	20	0.5	0.125	4:1	+15.81
OC-NE4	5	20	0.5	0.25	2:1	+20.18
OC-NE5	5	20	0.5	0.5	1:1	+20.49

57 Black, M.M.; Schwartz, H.M. The estimation of chitin and chitin nitrogen in crawfish waste and derived products. *Analyst* **1950**, *75*, 185–189.
